# Association between Y-Maze Acquisition Learning and Major Histocompatibility Complex Class II Polymorphisms in Mice

**DOI:** 10.1155/2018/6381932

**Published:** 2018-07-19

**Authors:** Ming Ru, Hui Liu

**Affiliations:** College of Medical Laboratory, Dalian Medical University, Dalian 116044, China

## Abstract

**Objective:**

To explore the association between the acquisition process in the Y-maze and H-2 class II polymorphisms in mice.

**Methods:**

Mice were trained for 5 consecutive days in the Y-maze. The value of the slope of the latent period was considered an indication for the acquisition process. A slope < 0 indicated learning during the training and a slope > 0 indicated no learning. The H-2 polymorphism was determined with PCR amplification, and the correlation between the alleles and the acquisition process was analyzed.

**Results:**

The overall percentage of mice that learned was 46.1%. The percentage of mice that had learned with MudoEb5 (37.9%) was significantly lower than that of mice without MudoEb5 (61.1%; P < 0.05). The percentage of mice that had learned with MudoEb7 (26.1%) was significantly lower than that of mice without MudoEb7 (51.9%; P < 0.05).

**Conclusions:**

The major histocompatibility complex (MHC) and other alleles may be involved in the acquisition process. There may be a biological basis for learning in mice.

## 1. Introduction

Animal learning and memory have been studied since the beginning of the 20th century. Learning and memory processes are crucial for an organism's capability to adapt to environmental changes. In humans, learning and memory are usually expressed through spoken or written language, whereas the cognitive function of animals can only be expressed through behavior. Learning and memory behavior can be displayed through several experimental models [1]. Most of the learning and memory models have been applied to study neurobiology [2, 3], physiology [4], pharmacology [5, 6], and gene engineering [7]. In recent years, more automated Y-maze applications based on a reduced instruction set computer microcontroller have been used to evaluate continuous spontaneous alternation in rats or mice [8, 9] which tend to learn actively or passively under the impact of some stressful stimuli [1, 2, 10]. Y-maze is a useful device to detect the animal's ability of learning and memory. Because of its simple structure and convenient operation, more and more animal experiments have adopted the Y-maze to explore the learning and memory of animals [11–13].

The major histocompatibility complex (MHC) is a set of genes found in vertebrates and plays a vital role in the adaptive immune response [14]. One of the significant features of MHC molecules is their complexity, as there are both polygenic-containing multiple genes and polymorphic-containing multiple variants of each gene [15]. MHC genes can be divided into three major categories, classes I, II, and III, all of which are known to play key roles in the healthy or diseased nervous system [16]. In the hippocampus, one or more MHC I genes are necessary for normal synapse density [17], synaptic transmission [18], synaptic plasticity [19], and some hippocampus-dependent formations of learning and memory [20]. Although the MHC I protein was traditionally thought to be absent from the surface of neurons [21, 22], multiple studies have detected it in hippocampal neurons [23], but these studies did not determine the gene expression of MHC I. Recent work also indicated that MHC I protein is expressed on the surface of axons and dendrites [24, 25].

The MHC of mice is named H-2, with genetic polymorphism loci being located on chromosome 17, mainly H-2 class II molecule I region, E subregion, especially the second exon of the *β* locus (H-2 Eb) [26, 27]. This region presents peptides to T-lymphocytes, which in turn induce an immune response to non-self-peptides [28]. From the centromeric side to the telomere side, four genetic regions are composed of K, I, S, and D [29], respectively, corresponding to I, II, III, and I of the MHC. Their polymorphisms are mostly concentrated in the MHC II molecules, which are composed of *α* and *β* chains [30]. Research has shown that immune cells are required for the regulation of the nervous system circuitry [31]. Early work mainly supports the presence of immune-nervous systems connections in animal models of infection and injury [32, 33], whereas a recent review also presented evidence for the involvement of the immune system in physiological neurobehavioral processes, indicating the importance of a functioning immune system for cognition [31]. Behavioral and neural plasticity are the most important aspects of brain functioning that are modulated by immune mechanism [32]. MHC class II is only expressed in some specific cell surfaces in the lymphoid tissue, such as specific antigen-presenting cells, including B-cells, macrophages, thymic epithelial cells, dendritic cells, and activated T-cells; the association between MHC class II molecules and learning/memory in animals is unknown; and both the immune system and nervous system have the properties of memory; therefore, this study analyzed whether the acquisition process of animals is related to MHC class II molecules.

## 2. Materials and Methods

### 2.1. Animals

A total of 102 KM (Come of Swiss) mice (n = 55) and BALB/c mice (n = 47) were obtained from the Laboratory Animal Center of Dalian Medical University. All mice were 6-week-old females and weighed 18~22g. KM and BALB/c mice were housed in separate rooms in a quiet environment at a controlled temperature of 23±2°C on a 12 h light/dark cycle (light on at 8 a.m.) with food (provided by the Animal Center of Dalian Medical University) and water* ad libitum*. Four animals were cohoused in plastic cages with wood chip bedding (cage dimensions: 260mm×180mm and 150mm high). Three days were allowed for acclimatization before the Y-maze tests. All animal experiments were conducted in accordance with the guidelines for the use of experimental animal and approved by the Animal Care and Use Committee of Dalian Medical University, which complies with the National Institutes of Health Guide for the Care and Use of Laboratory Animals. All experimental animals were humanely killed by decapitation after the last day of training.

### 2.2. Y-Maze Training

All 102 mice were trained daily at 3 p.m. for 5 days and divided into five batches. Each batch of mice included both KM and BALB/c. Y-maze tests were performed in a room with dim light. Training times and conditions were performed in a consistent manner and by the same individual who was blinded to the experimental batches. The mice of different strains were behaviorally evaluated in an alternating fashion in the testing period.

The ZH-MGY Y-maze video tracking and analysis system (Zheng Hua Biological Instrument and Equipment Co., Ltd., Anhui, China) was used, which consisted of three equally distributed arms (A, B, and C) at 120° angle. Each arm had an internal dimension of 470 mm× 160 mm and 460 mm high. Any of the three arms could be set as the starting zone through the computer, which was defined as a nonsafe zone after the experiment began. The remaining two arms were randomly divided into a safe zone without foot shock (current stimulation) and a nonsafe zone with foot shock by Y-maze video tracking and analysis system. A light bulb (62.8 lux) was placed at the end of each arm but was the only light source in the safe zone. The safe zone randomly alternated among the three arms. Mice were required to learn to avoid the electric shock induced by the light source in the study, which could minimize the influence of the odor [10].

The time taken to escape to the safe zone was recorded (escape latency); any escapes to nonsafe zones were regarded as erroneous, and the experiment continued until the mouse found the safe zone. The mean of ten training sessions was taken to calculate the average escape latency, which was automatically generated by the Y-maze video tracking and analysis system. A training session was completed when the mouse entered the safe zone with all four paws across the junction of the safe zone and the central zone (CZ) [34], the light was then turn off, and the safety zone became the starting zone of the next training session.

**Figure 1:**
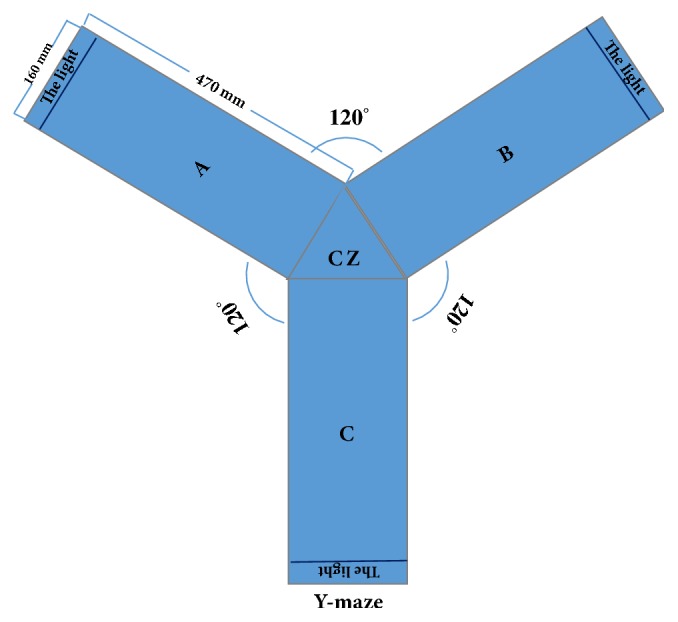
Y-maze apparatus and construction.

The settings of the experimental conditions were as follows: starting area: A zone; current: 0.6 mA; stimulation delay (time interval between the light source in the safe zone and the start of the current stimulation): 3 s; amount of test days: 5; test times: 10 times a day; stimulation intervals (time intervals between two training sessions): 30 s. Current stimulation was provided in the central zone. The Y-maze video tracking and analysis system consisted of three parts: a computer, a video camera, and the Y-maze apparatus made of acrylic (see Figure 1).

The Y-maze apparatus consisted of three arms (A, B, and C) at 120° angle, connected by a central zone (CZ). The electricity grid (made of stainless steel) was placed underneath each arm; the outer end of each arm had a light bulb providing the light source of the safe zone. A video camera was mounted vertically on top of the Y-maze and recorded the location of mice in real time.

Mice were acclimatized to the testing room before conducting the Y-maze and allowed to familiarize themselves with the maze for ~5 min before the training was carried out [35]. After 5 min, mice either returned to the preset starting area themselves or were put there by the experimenter to begin the experiment. Due to their innate preference for a dark environment, the conditioned reflex of the mice upon current stimulation was to escape to the other arm without the light source, but some trained mice actively avoided this conditioned reflex due to a current stimulus and fled into the safe zone that had no current but a light source. The escape latency of these mice decreased over time, indicating a successful learning process.

### 2.3. Analysis of Gene Polymorphism

After 5 days of training, we extracted genomic DNA from the tail tip of mice according to the manufacturer's instructions (TaKaRa MiniBEST Universal Genomic DNA Extraction Kit Ver.5.0, Takara Biology Technology Co., Ltd, Dalian, China). The peptide-binding region (PBR), encoded by exon 2 of MHC II b, is a highly polymorphic region in vertebrate [26, 27, 36]. The GenBank accession numbers in this experiment were U88914 and U88916. Two primers for MudoEb5 and MudoEb7 were designed and synthesized [37] (Takara Biology Technology Co., Ltd., Dalian, China): MudoEb5 forward primer: 5′-GGA GAA CCT GCG CTT CGA C-3′; reverse primer: 5′-TCT TTG ATC CAG GAA CTC CGG-3′; MudoEb7 forward primer: 5′-CCA TGG TTT TTG GAA TAC TCT ACA-3′; reverse primer: 5′-GCA GGT TCT CCT CCA GGT T-3′. Using a 25 *μ*L reaction system (1 *μ*L of gene template, 1 *μ*L of gene forward and 1 *μ*L of gene reverse primers, 12.5 *μ*L of 2×Power Taq PCR Master Mix, and 9.5 *μ*L of ddH_2_O), the reaction conditions included initial denaturation (94°C, 2 min), denaturation (94°C, 30 s), annealing (62°C, 30 s), and extension (72°C, 30 s). A total of 35 cycles were performed. MudoEb5 and MudoEb7 gene regions were amplified for each DNA specimen. The results of the agarose gel electrophoresis were analyzed with a UVPC-80 gel imaging system (UCP Inc., San Jose, CA, USA).

### 2.4. Statistical Analysis

The training results were recorded by Y-maze video tracking and analysis system. The horizontal coordinate represents the number of days; the longitudinal coordinate represents the escape latency. The slope was calculated from the average escape latencies measured on days 3, 4, and 5 and was used as an indicator of learning (a slope < 0 indicates learning; a slope > 0 indicates no learning). The quartiles were used to observe the median of all slopes. Pearson's chi-square test and logistic regression were used to analyze the relationship between the two mouse strains, two genotypes, and slopes.

## 3. Results

### 3.1. Escape Latency after 5 Consecutive Days of Training

**Figure 2:**
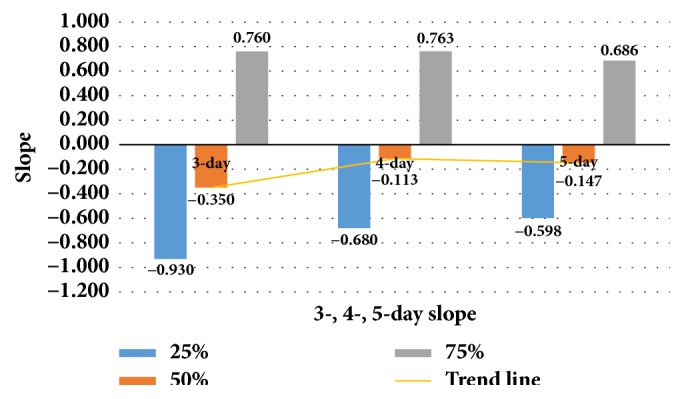
Comparative analysis of 3-, 4-, and 5-day slopes as learning indices.

Table 1 shows the medians of the 3-, 4-, and 5-day slopes. The median comparison analysis revealed that the 3-day slope of mice was the best, meaning that the percentage of the acquisition learning was highest from the first day of training to the third day of training for BALB/c. However, there was no significant progress in learning and memory during the following 2 days of training (see Figure 2). A total of 25% of KM mice and 50% of BALB/c mice demonstrated learning (slope < 0; see Table 1). Learning and memory performance was better in BALB/c than KM mice. A slope > 0 indicates no learning.

“0” was considered as the cut-off value. After training for 3 days, the training continued and 4- and 5-day slopes were calculated. The absolute value of the median slope showed a downward trend. The acquisition of learning reached a plateau after day 3; therefore, the 3-day slope was chosen for the following analysis.

### 3.2. Comparison and Analysis of Acquisition Learning Based on the 3-Day Slope

Based on the 3-day slope, we analyzed the proportion of learning vs. nonlearning mice between the two strains. During the 3-day training process, 46.1% of mice had learned (KM: 34.5%, BALB/c: 59.6%). The learning and memory of BALB/c mice were better than those of KM mice. Pearson's chi-square test showed a significant difference between the strains (P < 0.05; see Table 2).

### 3.3. Relationship between Acquisition and H-2 Polymorphism in Mice

**Figure 3:**
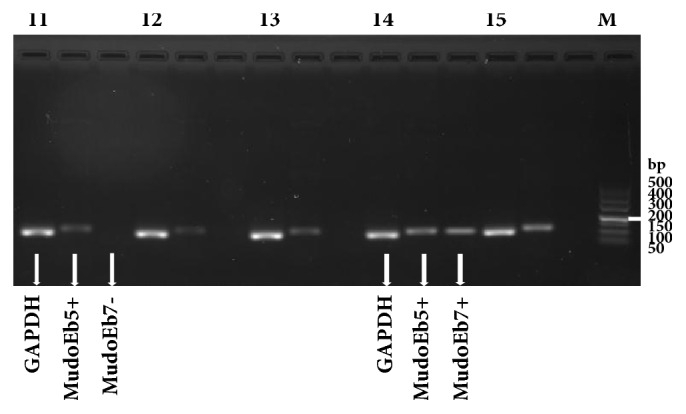
PCR amplification products of MudoEb5 and MudoEb7.

Polymorphic loci were amplified with Polymerase Chain Reaction (PCR); the resulting bands are shown in Figure 3. Three pairs of primers were amplified in each specimen.* GAPDH *was used as a quality control of the process of sample extraction and PCR; its product was 90 bp. MudoEb5 was the first target gene; its product was 109 bp. MudoEb7 was the second target gene; its product was 103 bp. The marker on the right denotes 500 bp.* GAPDH* was amplified as an internal reference in all five specimens, demonstrating that the amplification system and conditions were fulfilled. Only MudoEb7 was amplified in specimen 14; all other specimens were amplified MudoEb5. The relationship between the two alleles and the training results (3-day slope) can be found in Table 3. The percentage of mice that learned with MudoEb5 and MudoEb7 was 37.9% and 26.1%, respectively. The percentage of mice that had learned without MudoEb5 and MudoEb7 was 61.1% and 51.9%, respectively. These results suggest that to a certain extent MudoEb5 and MudoEb7 were associated with learning and memory. The percentage of mice that had learned with two polymorphism loci was lower than those without two polymorphic loci, indicating that two polymorphic loci had affected learning and memory.

### 3.4. Relationship between Polymorphic Loci, Strain, and Learning and Memory by Logistic Regression

The differences in learning and memory were both related to genetic polymorphisms and to the strain of the mice (see Table 4). According to the three steps of the regression analysis, although the two polymorphic loci were related to learning and memory, the most important factor was the strain. Therefore, other alleles may be involved in the formation of learning and memory. The genetic difference between the two strains may contribute to their learning and memory ability.

## 4. Discussion

Two different mouse strains were used in this study for abundant polymorphism of H-2. All 102 mice were randomly selected in the experiment, no animals were excluded from the study, and all training sessions were analyzed.

Mice learn and form memory in a short period of time, and the formation of learning and memory is not only related to the hippocampal and the marginal division of striatum [38], but also related to genetic polymorphisms (see Table 3). Differences in training results between the two different strains of mice were observed, as BALB/c mice performed better than KM mice (see Table 2). There were both intra- and interspecies differences in learning and memory.

After 3 days of Y-maze training, the acquisition of learning reached a plateau. Learning and memory abilities differed between mice strains. Therefore, mice of different strains could be selected to establish a learning model and to study the molecular biological mechanism of animal learning. Mice with two polymorphic H-2 loci performed worse, suggesting that the presence of two polymorphic loci may impair learning and memory formation. Multiple alleles are likely involved in the learning process of mice. These results provide the basis for further analysis of the ability of animal learning and memory from the perspective of genetic polymorphism.

One limitation of this study is the foot shock application during the experiment, which may elicit different degrees of tolerance and anxiety in mice. This behavior may be displayed as climbing and crouching, which could affect the data interpretation. In this study, mice were trained for 5 consecutive days, and then 3-, 4-, and 5-day slopes were calculated accordingly. It was found that the 3-day slope was most appropriate, better than 4/5-day slope. Mice would feel anxiety because of the foot shock [39] during a long time; thus 3-day slope used in this study could avoid this influence to some extent.

In this study, we found a significant learning difference between two strains, which warrants further research characterization of learning and memory abilities among mouse strains. Learning and memory in mice are influenced by MHC polymorphisms, and complex processes controlled by multiple genes are likely involved.

## 5. Conclusion

H-2 class II gene polymorphism was associated with learning and memory in mice. MHC and other alleles may be involved in the acquisition process. This study analyzed the acquisition process from the perspective of gene polymorphisms, which lays a foundation for a further understanding of the relationship between learning and memory and gene polymorphisms.

## Figures and Tables

**Table 1:** Comparative analysis of 3-, 4-, and 5-day slopes using the quartiles (25th, 50th, and 75th).

Strain	3-day slope	4-day slope	5-day slope
KM mice	−0.945;0.300;2.990	−1.020;0.354;1.460	−0.621;0.595;2.255

BALB/c mice	−0.930;−0.350;0.760	−0.680;−0.113;0.763	−0.598;−0.147;0.686

**Table 2:** Relationship between acquisition and strain.

Strain	N	L	L%	*χ* ^2^	P
KM mice	55	19	34.5%	6.390	0.011
BALB/c mice	47	28	59.6%		

TOTAL	102	47	46.1%	-	-

3-day slope: < 0 indicates learning; >0 indicates no learning. N: sample size; L: number of mice that have learned.

**Table 3:** Comparison of MudoEb5 and MudoEb7 gene polymorphisms distributed in the mice analyzed by Pearson's chi-square test.

Allele	N	L	L%	*χ* ^2^	P
MudoEb5+	66	25	37.9%	5.060	0.024
MudoEb5−	36	22	61.1%		

MudoEb7+	23	6	26.1%	4.777	0.029
MudoEb7−	79	41	51.9%		

3-day slope: < 0 indicates learning and >0 indicates no learning. N: sample size; L: number of mice that have learned.

**Table 4:** Parameter estimation and test results of logistic regression.

Allele	B	S.E.	Exp (B)	P
MudoEb7	Removed in step 1	0.608	1.746	0.359
MudoEb5	Removed in step 2	0.490	1.746	0.255
Strain	−1.027	0.411	0.358	0.012

MudoEb5 and MudoEb7 gene polymorphism factors were excluded from the regression equation.

## Data Availability

The data used to support the findings of this study are included within the article.
